# Supplementary material for: What Is Wrong with Frankenia nodiflora Lam. (Frankeniaceae)? New Insights into the South African Sea-Heaths
Source: Plants (Basel). 2023 Jul 13;12(14):2630. doi: 10.3390/plants12142630 (PMC10383076; doi:10.3390/plants12142630)
Supplement: Supplementary file 1 [file plants-12-02630-s001.zip › plants-2435000-supplementary.pdf]

What is wrong with *Frankenia nodiflora* Lam. (*Frankeniaceae*)? Restoration of a misunderstood name and description of two new related perennial species from South Africa

Manuel B. Crespo [1,*], María Ángeles Alonso [1], Mario Martínez-Azorín [1], José Luis Villar [1] and Ladislav Mucina [2,3]

1 Departamento de Ciencias Ambientales y Recursos Naturales (dCARN), Universidad de Alicante, P.O. Box 99, ES-03080 Alicante, Spain; ma.alonso@ua.es (M.M.A.); mmartinez@ua.es (M.M.-A.); jose.villar@ua.es (J.L.V.)

2 Iluka Chair in Vegetation Science and Biogeography, Harry Butler Institute, Murdoch University, 90 South Street, Murdoch WA 6150, Perth, Australia; ladislav.mucina@murdoch.edu.au

3 Department of Geography and Environmental Studies, Stellenbosch University, Private Bag X1, Matieland 7602, Stellenbosch, South Africa

* Correspondence: crespo@ua.es

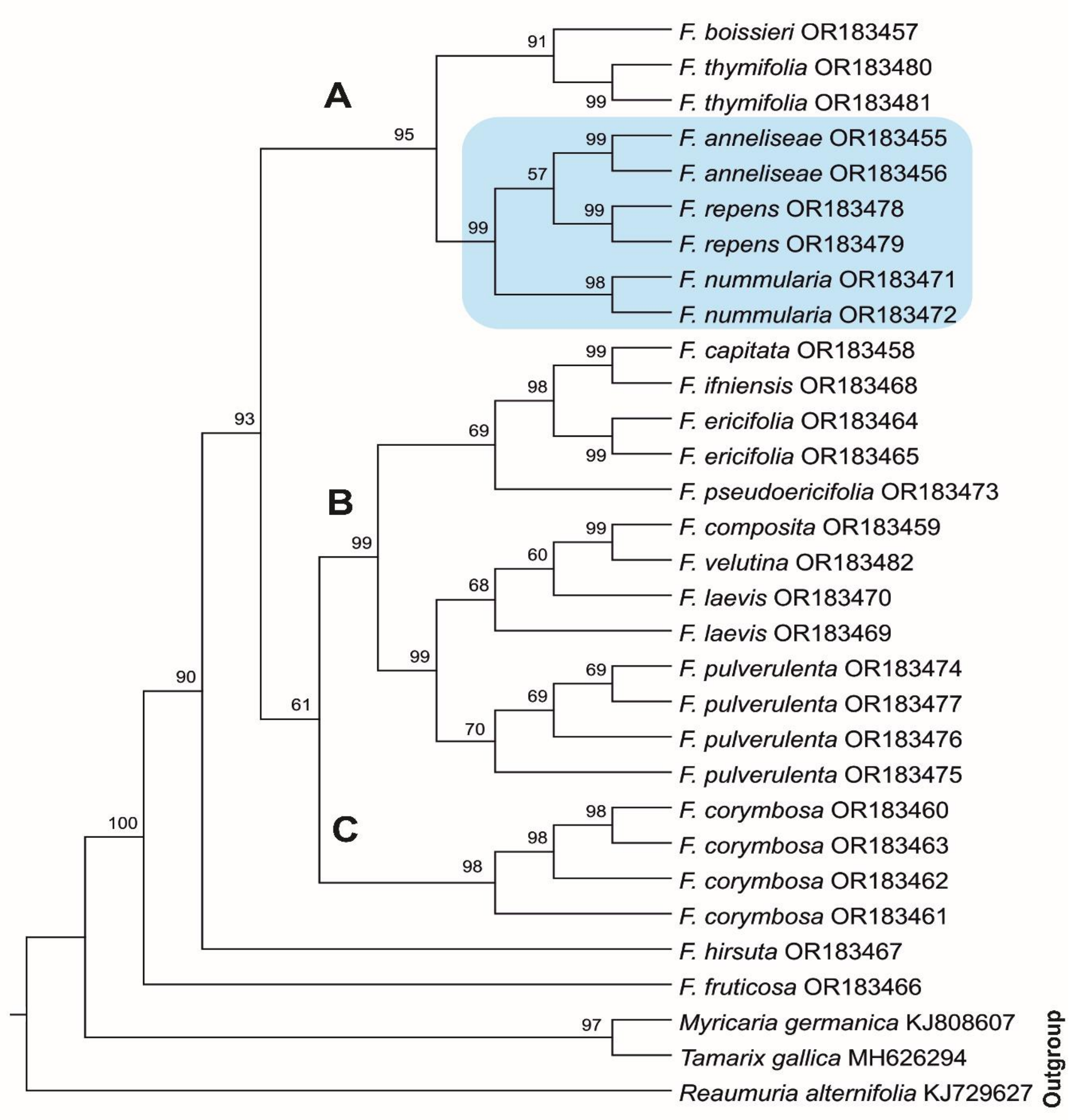

Figure S1. Maximum Parsimony (MP) 50% consensus phylogenetic tree of *Frankenia* accessions from ITS nuclear DNA sequences. Nine most parsimonious trees were obtained with a tree length (TL) of 516 steps, a consistency index (CI) of 0.769 and a retention index (RI) of 0.863. Members of the "*Frankenia repens* group" are highlighted in clade A. The MP tree was obtained using the Subtree-Pruning-Regrafting (SPR) algorithm with search level 1, in which the initial trees were obtained by the random addition of sequences (10 replicates) Numbers above branches indicate bootstrap percentage (BP) values obtained in the 50% consensus-tree after 10,000 replicates. GenBank codes are shown after each taxon/accession name.

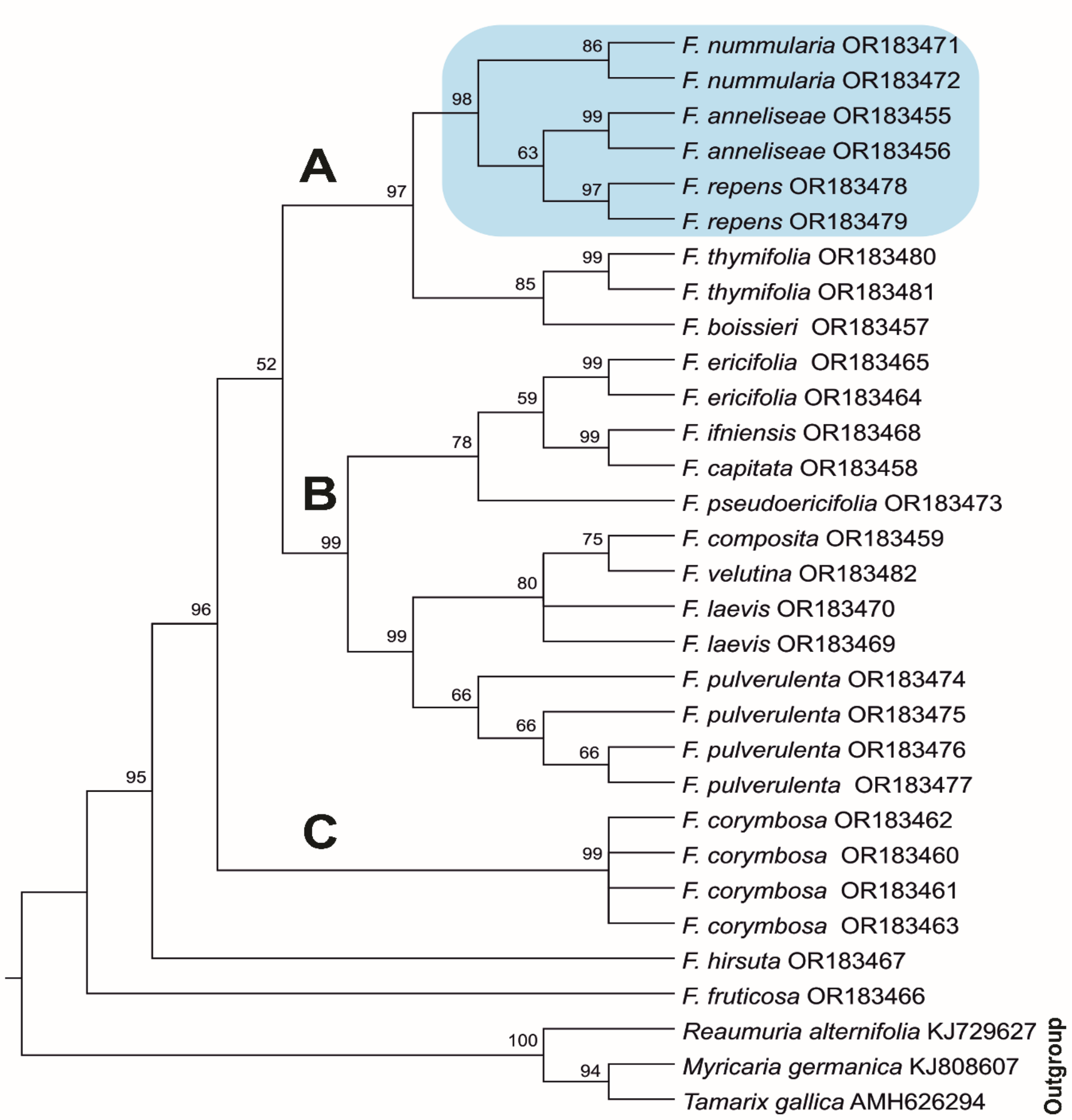

Figure S2. Neighbour Joining (NJ) phylogenetic tree of *Frankenia* accessions from ITS nuclear DNA sequences. Members of the "*Frankenia repens* group" are highlighted in clade A. Numbers above branches indicate percentage of replicate trees in which the associated taxa clustered together in the bootstrap test (10,000 replicates). The evolutionary distances were computed using the Kimura 2-parameter method. GenBank codes are shown after each taxon/accession name.
